# E3 Ubiquitin Ligases in Gammaherpesviruses and HIV: A Review of Virus Adaptation and Exploitation

**DOI:** 10.3390/v15091935

**Published:** 2023-09-15

**Authors:** Jessica Oswald, Mathew Constantine, Adedolapo Adegbuyi, Esosa Omorogbe, Anna J. Dellomo, Elana S. Ehrlich

**Affiliations:** Department of Biological Sciences, Towson University, Towson, MD 21252, USA

**Keywords:** gammaherpesvirus, ubiquitin, rhadinovirus, human herpesvirus-4, human herpesvirus-8, Epstein–Barr virus, HIV

## Abstract

For productive infection and replication to occur, viruses must control cellular machinery and counteract restriction factors and antiviral proteins. Viruses can accomplish this, in part, via the regulation of cellular gene expression and post-transcriptional and post-translational control. Many viruses co-opt and counteract cellular processes via modulation of the host post-translational modification machinery and encoding or hijacking kinases, SUMO ligases, deubiquitinases, and ubiquitin ligases, in addition to other modifiers. In this review, we focus on three oncoviruses, Epstein–Barr virus (EBV), Kaposi’s sarcoma herpesvirus (KSHV), and human immunodeficiency virus (HIV) and their interactions with the ubiquitin–proteasome system via viral-encoded or cellular E3 ubiquitin ligase activity.

## 1. Introduction

Ubiquitination is a post-translational modification most associated with targeting proteins for proteasomal degradation but is also a key regulator of other non-proteolytic cellular processes. Proteins can be monoubiquitinated via the addition of the 76-amino acid ubiquitin molecule to a lysine (K) in the substrate protein. They can also be polyubiquitinated via the addition of polyubiquitin chains with diverse intraubiquitin K linkages or linkages via the N-terminal methionine. K48-linked and K63-linked polyubiquitin chains are best characterized by their roles in protein degradation and signaling complex assembly, respectively. Additional functions of polyubiquitin chains with any combination of the 7-lysine and N-terminal methionine linkages, including mixed and branched chains, and modification with the small ubiquitin-like modifier (SUMO) or other modifications such as phosphorylation together make up a ubiquitin code that is involved in the regulation of diverse cellular processes from metabolism to DNA repair to autophagy, to name a few [1]. Ubiquitination is mediated via the activities of ubiquitin-activating enzymes (E1), ubiquitin-conjugating enzymes (E2), and ubiquitin ligases (E3), which work in balance with deubiquitinating enzymes (DUBs). E3 ubiquitin ligases are essential for substrate specificity and are the focus of this review [2].

Viral manipulation of the ubiquitin–proteasome system (UPS) alters the host’s ubiquitome, targeting proteins with diverse functions from host restriction factors to antiviral proteins. Viral targeting of tumor suppressors and oncogenes can promote oncogenesis, highlighting the importance of these interactions in the transformation ability of oncoviruses. Roughly 13% of global cancer cases can be attributed to infectious diseases with viruses accounting for most of these cases [3]. There are seven viruses associated with oncogenesis: Epstein–Barr virus (EBV), human papillomavirus (HPV), Kaposi’s sarcoma-associated herpesvirus (KSHV), hepatitis B virus (HBV), hepatitis C virus (HCV), human T-cell lymphotropic virus-1 (HTLV-1), and Merkel cell polyoma virus (MCV). Human immunodeficiency virus (HIV) is often grouped with cancer-causing viruses due to the immune system’s damaging effects of infection, which result in an increased risk of developing cancer, especially in the context of co-infection with the above viruses. These oncoviruses are highly variable, representing both DNA and RNA viruses from diverse families. Each of these oncoviruses either express a virally encoded ubiquitin ligase or interact with cellular ubiquitin ligases. Here, we will focus on gammaherpesviruses (EBV and KSHV) and HIV and their interactions with both viral and cellular E3 ubiquitin ligases.

## 2. Gammaherpesviruses

The Herpesviridae family consists of large linear double-stranded DNA viruses, which are able to establish a latent infection wherein the viral genome is maintained within the host nucleus as extrachromosomal circular episomes [4]. There are eight known human herpesviruses, which can be categorized into three subfamilies: α, β, and γ [4]. Gammaherpesviruses establish a lifelong latent infection with periods of lytic replication primarily in lymphoid cells, but are also known to replicate in nonlymphoid cells [4]. KSHV genomes and transcripts have been detected in B cells, endothelial cells, monocytes, keratinocytes, and epithelial cells [5]; EBV primarily infects B cells and epithelial cells, but has also been associated with malignancy in T cells and NK cells [6,7]. EBV and KSHV are clinically associated with very different diseases, but they share oncogenic capabilities. Additionally, both viruses have evolved to either encode their own or exploit host ubiquitin ligases to manipulate the cellular UPS, which aids in infection and contributes to oncogenesis [2].

### 2.1. Epstein–Barr Virus

Epstein–Barr virus or human herpesvirus-4 (EBV/HHV-4), is a gammaherpesvirus with a ~172 kb DNA genome and infects more than 95% of the world population [8]. Globally, primary infection is typically contracted via saliva during infancy and is often asymptomatic; however, in more developed countries, primary infection frequently occurs after childhood [8]. During primary infection, most people are asymptomatic or experience flu-like symptoms, although individuals infected as young adults may experience the more serious disease known as infectious mononucleosis, for which EBV is the causative agent [8]. EBV primarily establishes latent infection in B cells but can also infect T cells and epithelial cells and is subsequently associated with multiple lymphoproliferative and non-lymphoproliferative malignancies, including Burkitt’s and Hodgkin’s lymphoma and gastric and nasopharyngeal carcinoma (NPC) [4]. EBV establishes infection in the oropharynx where it preferentially targets B lymphocytes by binding the major viral envelope glycoprotein gp350 to the CD21 surface B cell receptor and binding gp42 to HLA-class II as a co-receptor [9]. Infection in other cell types like epithelial cells, occurs as well, but is less efficient and via separate pathways, not as well understood [10]. EBV can transform B cells into immortalized latently infected lymphocytes that reside in lymphoid tissues and become part of the B cell memory pool promoting lifelong infection and that contributes to lymphomagenesis and epithelial carcinogenesis [11,12]. 

EBV has a biphasic lifecycle with two distinct stages: latency and lytic replication. During latency, select genes are expressed that contribute to the EBV-mediated transformation of B cells. Uniquely, EBV displays three types of latent infection known as latency I, II, and III. The dominant mode of infection, latency III is characterized by the transformation of B cells to latently infected lymphoblastoid cell lines (LCL) that maintain a lifelong and usually asymptomatic infection. EBV-transformed LCLs carry several extrachromosomal copies of the viral episome that allow for the expression of a select number of genes. LCLs express nine viral gene products known as latent proteins, including six EBV nuclear antigens (EBNAs 1, 2, 3A, 3B, 3C, and LP) and three latent membrane proteins (LMPs 1, 2A, and 2B) [13]. Particularly, latent proteins EBNA2, EBNA3C, and LMP1 play a key role in inducing LCL-like phenotypic transformation of B cells [14]. During initial infection, EBV employs a “growth program” that is likely required to expand the pool of virally infected cells to increase the likelihood of transformed lymphocytes integrating into the memory population. This growth program is closely associated with latency III, making this latent type particularly dangerous for immunocompromised individuals and transplant recipients [15].

#### 2.1.1. Latency

Additional types of latency display a more restricted expression of viral gene products. Latency II utilizes latent proteins EBNA1, LMP1, LMP2A, and LMP2B and is associated with the development of NPC and NK/T cell lymphoma, whereas latency I is associated with Burkitt’s lymphoma and gastric cancer. However, the only latent viral gene product consistently observed during latency I is EBNA1. All EBV latently infected cells also display abundant expressions of noncoding EBER RNAs and transcripts from the BamHI-A region of the viral genome known as BART transcripts that are precursors to BART miRNA [13,16]. Regardless of modality, successful latent infection persists due to the ability of latent proteins to modify B cell receptor (BCR) signaling and evade the immune response via manipulation of host cellular pathways such as the UPS [8,17]. Of the latently expressed genes, LMP1, LMP2A, EBNA1, and EBNA3C have a demonstrated ability to hijack various E3 ubiquitin ligases to modulate BCR signaling and promote latency (Table 1) [17]. Like all herpesviruses, the alternate life stage of EBV is lytic replication. This life cycle is characterized by an increase in viral lytic gene expression promoting viral replication [13]. This article will first focus on latent proteins and return to lytic replication.

Latency types II and III both consistently utilize latent membrane proteins LMP1, LMP2A, and LMP2B. These proteins are important for regulating B cell activation and apoptosis. Together, they can act as a so-called rescue program that promotes the survival and maturation of lymphoblasts to integrate into the memory B cell population [44]. Particularly, LMP1 is essential for subverting BCR communication by mimicking CD40 signaling and activating the nuclear factor kappa B (NF-κB), mitogen-activated protein kinase (MAPK), JAK/STAT, phosphatidylinositol 3-kinase (PI3K), and SUMOylation pathways [13,45]. Due to this, LMP1 plays an essential role in the transformation of B lymphocytes to LCLs in latency III. As mentioned previously, EBV employs a growth program during the initial infection causing latency III to be associated with autonomous B cell proliferation. This becomes hazardous for EBV as increased rates of replication can be lethal to the host. To manage this, some proteins secreted during the growth program enhance immunogenicity making infected cells easy targets for T cell-mediated immune responses. LMP1 is one of the main drivers of this phenotype by acting as a master switch that influences proliferation, immunogenicity, and survival [15]. In addition, LMP1 is the only oncogenic EBV protein. Transfection of LMP1 in both mice or human fibroblasts and epithelial cells resulted in tumorigenicity [46,47,48] and transfection in transgenic mice contributed to hyperproliferation and lymphomas [49,50]. LMP1 is typically a short-lived protein [51] that is degraded by ubiquitin-dependent proteolysis [52]. Uniquely, the ubiquitin site of LMP1 is likely located in the N terminus as ubiquitin modification of this region results in full stabilization [15].

#### 2.1.2. LMP1

While other reviews have discussed mechanisms associated with LMP1 activity, we will limit our discussion to interaction with host ubiquitin ligases. LMP1 regulates interactions between EBV-infected cells and the environment by recruiting tumor necrosis factor receptors such as tumor necrosis factor receptor-associated factor (TRAF) proteins and TRADD to activate cellular pathways [15]. LMP1 activates the NF-κB signaling pathway via interaction with TRAF6, resulting in the K-63-linked polyubiquitination of IKK [18,19,20,21]. This interaction is mediated by the ubiquitin adaptor/sensor p62/SQSTM1, which is required for TRAF6 ubiquitination [22]. LMP1 does not target TRAF2 or TRAF3 for proteasomal degradation resulting in their constant activation during latency [53]. Consistent activation of NF-kB signaling is required for latency as it represses BZLF1-induced lytic reactivation. To counteract this repression, signaling is turned off via the DUB activity of BPLF1 [54]. Immunoprecipitation experiments have also revealed that TRAF6 mediates LMP1 signaling to p38 MAPK via a TRADD-dependent pathway [18]. The recruitment of TRAF6 and subsequent activation of p38 MAPK relied on an intact PxQxT motif located in the C terminal activator region 1 (CTAR1) and tyrosine 384 in CTAR2 of LMP1 [18].

Additionally, linear ubiquitin assembly complex (LUBAC)-mediated ubiquitination has been an active topic of research due to increasing evidence of its role in activating NF-κB signaling. Previous studies demonstrated that the combined knockdown of the critical LUBAC components, RNF31 and RBCK1, blocked NF-κB activation by LMP1 [55]. A more recent study demonstrated that LMP1 and IRF7 interact directly with RNF31 leading to LUBAC-mediated ubiquitination of NEMO and IRF7 to promote NF-κB signaling and inhibit IRF7 transcriptional activity [23]. Data also suggests that RNF31 is a downstream transcriptional target of LMP1 further linking EBV and LUBAC [23]. These findings demonstrate that LUBAC is essential for the full activation of NF-κB signaling and regulation of IRF7 transcriptional activity [23].

It was previously discovered that p53 is accumulated in EBV-infected tumors, most commonly in NPC [56,57,58]. LMP1 promotes p53 accumulation by phosphorylation [59,60], as well as two distinct ubiquitin modifications [24]. Interactions with the E3 ligases, MDM2 and TRAF2, mediated by LMP1 modulates K48-linked and K63-linked polyubiquitination of p53, respectively [24]. LMP1 suppresses MDM2 addition of K48-linked polyubiquitin chains to p53 due to associated p53 phosphorylation at Ser20, enhancing p53 accumulation and stability [24]. LMP1 also aids TRAF2 binding with p53 to enhance K63 ubiquitination and increase p53 accumulation thereby suppressing apoptosis and cell cycle arrest [24]. In addition, a screen utilizing co-immunoprecipitation followed by mass spectrometry revealed 19 candidate E3 ligases that LMP1 can interact with including a chaperone-dependent ligase, CHIP [25]. CHIP is known to interact directly with RIG-I to induce its degradation via K48-linked polyubiquitination. Therefore, this study suggests that CHIP recruitment induces observed RIG-1 degradation and subsequently prevents IFN-β expression [25]. Together, these interactions provide further evidence of LMP1′s role in downregulating the antiviral response via manipulation of the UPS during latency.

#### 2.1.3. LMP2A

The triggering of BCR signaling is one of the main contributing factors that drives EBV-infected cells to lytic replication [61]. LMP2A regulates the stimulation of BCR signaling by interfering with BCR kinases [62,63]. LMP2A has an N-terminal intracellular domain that mimics BCR signaling and a C-terminal region that contributes to blocking BCR signaling [64]. The N-terminus contains eight tyrosine residues that upon phosphorylation can bind with the SH2 domains of BCR tyrosine kinase Syk and Src family kinase Lyn [65]. The N-terminus of LMP2A also has two PY binding motifs (PPPPY) that recruit and bind NEDD4 family ubiquitin ligases, specifically AIP4 and WWP2, via their WW domain [26,27,28,29]. One result of this is that LMP2A is ubiquitinated by the homologous to the E6-AP carboxyl terminus (HECT) domain of these ligases [26,27,28,29]. However, these findings also support the observation that LMP2A enhances the ubiquitination of both Lyn and Syk and the destabilization of Lyn in a NEDD4 family ligase-dependent manner, likely by acting as a scaffold for molecular interactions [30]. Unlike Lyn, the ubiquitination of Syk does not result in a change in turnover [30]. Rather, phosphorylation of Syk has demonstrated importance for LMP2A to promote constitutive activation of Syk substrate, SLP-65 [31]. This induces the recruitment and formation of a ternary protein complex including E3 ligase Cbl, nucleotide exchange factor C3G, and the proto-oncogene CrkL [31]. Cbl-b ubiquitinates Syk and contributes to the negative regulation of BCR signaling [32].

In addition to hijacking ubiquitin ligases, there is evidence of LMP2A being targeted by E3 ligases to downregulate LMP2A signaling as part of the host defense. Itch and c-Cbl have been observed to interact with LMP2A to induce ubiquitination and degradation and thereby modulate LMP2A activity [27,33,66]. These interactions provide further evidence that LMP2A is indispensable for EBV-mediated B cell transformation and establishing latent infection.

#### 2.1.4. EBNAs

EBNAs are essential for latency by promoting the conversion of primary B lymphocytes to lymphoblastoid cells and regulating host transcription factors [64]. Interestingly, EBNA1 is the only latent protein to be consistently observed in all latency programs. Its necessity is highlighted by its function. EBNA1 binds the dyad symmetry and repeat sequences in the origin of plasmid replication (oriP) of the viral genome. This allows EBNA1 to regulate the replication of the viral episomes alongside the cellular DNA and maintain their separation during cell division. EBNA1 is also a transcription regulator that acts on the promoters Wp, Cp, and its own promoter Qp [67]. Several functional domains of EBNA1 such as nuclear localization signal and DNA binding reside in the C-terminal domain. Whereas the N-terminal domain is comprised of a long repetitive sequence of Gly and Ala (GAr) residues only that vary in length between EBV isolates [68]. It is suggested that GAr makes endogenous EBNA1 resistant to processing by the proteasome for MHC class I presentation. This was demonstrated when the removal of the GAr region resulted in increased protein turnover and enhanced presentation of epitopes [69,70]. Additionally, EBNA1 has recently been observed to recruit an E3 ligase to aid its role in maintaining the EBV episome and regulating transcription. A SIM motif has been identified in EBNA1 (EBNA1^SIM^) that allows it to interact with the SUMO-modified KAP1 and STUB1, which has E3 ligase capabilities [34]. EBNA1 has been observed to recruit STUB1 to inhibit the SUMO modification of KAP1, which has implications for the regulation of latency and lytic reactivation [34].

There are several EBNA3 genes tandemly located in the viral genome upstream of promoters Wp and Cp. Particularly, EBNA3A and EBNA3C have been shown to be indispensable for B cell immortalization [71]. Both are known to down-regulate p16 in LCLs [72]. Specifically, EBNA3C displays the ability to facilitate the degradation of cell cycle regulatory proteins such as retinoblastoma (Rb) protein and p27 by targeting the E3 ligase SCF^skp2^ complex [35,36,37]. EBNA3C recruits SCF^skp2^ to increase ubiquitination and degradation of p27 leading to enhanced cyclin A-dependent kinase activity [35,36,37]. This interaction provides evidence of a mechanism for EBV-mediated cell cycle regulation [35,36,37]. Similarly, EBNA3C can recruit SCF^skp2^ to target Rb for proteasomal degradation [35]. Additionally, EBNA3C has demonstrated intrinsic deubiquitination capabilities and has been found to deubiquitinate itself and MDM2. Interactions between EBNA3C and Mdm2 stimulate the E3 ligase activity of MDM2 resulting in increased ubiquitination and degradation of p53 [38].

#### 2.1.5. Lytic Cycle

The alternate state of EBV infection is lytic replication. Lytic replication periodically occurs in transformed lymphocytes and rarely in infected epithelial cells. The physiological triggers of lytic replication remain unclear but some chemical factors such as TPA, calcium ionophore, sodium butyrate, anti-Ig and TGF-β are known to activate immediate-early gene expression [73]. Replication is initiated by the simultaneous expression of immediate-early genes BZLF1 and BRLF1 that encode transactivator proteins called ZEBRA including Zta (BZLF1) and Rta (BRFL1). BZLF1 then can activate some early lytic genes and there is an increase in transcription and cell proliferation to support the production of infectious viral progeny. This stage therefore results in cell death and the release of virions that may infect naïve cells within the host [8]. EBV has evolved molecular mechanisms to manipulate E3 ubiquitin ligases and the UPS to promote lytic replication (Table 1) [17].

#### 2.1.6. BFRF1

During lytic replication, the viral DNA is replicated and packaged into capsids that are then transported from the nucleus to the cytoplasm for tegumentation and maturation [13]. BFRF1 is a protein expressed during lytic reactivation that is a homolog of the herpes simplex virus 1 (HSV1) UL34 that plays a role in the nuclear egress complex that modulates the nuclear envelope to allow the transport of nucleocapsids [74]. BFRF1 has demonstrated the ability to interact with the cellular endosomal sorting complex required for transport (ESCRT) machinery that is known to be used by other enveloped viruses to aid with budding and viral release [75]. Uniquely, BFRF1 appears to be the first protein to use ESCRT machinery via recruitment of ESCRT adaptor protein Alix demonstrated by Alix colocalizing with BFRF1 in the nucleus. Following the dominant negative expression of ESCRT machinery, EBV virion release and retention of BFRF1 at the nucleus was blocked. Additionally, the inhibition of ESCRT abolished BFRF1-induced vesicle formation, which led to the accumulation of viral DNA and capsids in the nucleus of EBV-replicating cells. This suggests that ESCRT is utilized by BFRF1 to modulate the nuclear envelope for viral egress [74]. Recent studies have shed light on the mechanism used by BFRF1 to modulate ESCRT machinery and nuclear vesicles. It is established that the ESCRT system is modulated by NEDD4 E3 ligases such as Itch. BFRF1 is capable of binding serval NEDD4 ubiquitin ligases and is ubiquitinated itself. Removal of lysine ubiquitin sites resulted in disruption of BFRF1-induced vesicle formation and viral replication. Of the NEDD4 ligases BFRF1 associates with, it preferably binds Itch. Itch is able to bind with both Alix and BFRF1. Itch and Alix are both required for the BFRF1 induced formation of nuclear vesicles [41,42].

#### 2.1.7. BRLF1

In addition to NEDD4 ligases, a few lytic proteins have demonstrated the ability to interact with E3 ligase TRIM5α, which is known to be involved in the restriction of retroviral infection. As mentioned previously, BRLF1 (Rta) is known as an immediate-early lytic protein that promotes the transcription of viral lytic genes. A recent study demonstrated by GST-pulldown and coimmunoprecipitation that BRLF1 and TRIM5α interact via the N terminal domain of BRLF1 and the RING domain of TRIM5α. TRIM5α was also shown to promote ubiquitination of BRLF1 [43]. This is thought to be a mechanism by which EBV is able to maintain latency and mediate lytic activation.

#### 2.1.8. BPLF1 and BGLF2

Additionally, EBV encodes a viral deubiquitinating enzyme, BPLF1 that is a late lytic gene associated with the modulation of the host immune response. A study using coimmunoprecipitation and mass spectrometry to identify cellular targets of viral enzymes demonstrated that BPLF1 promotes the assembly of a trimolecular complex that includes the E3 ubiquitin ligase, TRIM25. TRIM25 in the presence of BPLF1 dimerizes and autoubiquitinates preventing subsequent ubiquitination of RIG-1 [39]. This results in inactive RIG-1 and therefore inhibits the downstream type-1 interferon response [39].In a recent study, the EBV tegument protein, BGLF2, was found to associate with the E3 ubiquitin ligase, Cullin 1 [40]. This allowed for recruitment of Cullin 1 to STAT2 resulting in STAT2 degradation and subsequent interruption of the innate immune response [40].

### 2.2. Kaposi’s Sarcoma Herpesvirus

Kaposi’s sarcoma-associated herpesvirus or human herpesvirus-8 (KSHV/HHV-8) is the etiological agent of numerous malignancies, including Kaposi’s sarcoma (KS), primary effusion lymphoma (PEL), and multicentric Castleman’s disease (MCD) [76]. As a gammaherpesvirus, KSHV exhibits a biphasic life cycle and establishes lifelong latent infections in its hosts. During latency, KSHV only expresses a handful of viral genes including the latency-associated nuclear antigen (LANA), viral cyclin (vCYC), and the viral FLICE inhibitory protein (vFLIP) [76]. The shunting of the virus from latency into the lytic state is influenced by host physiological and environmental conditions, including hypoxia, immune suppression, viral co-infection, and oxidative stress [76,77]. Conditions such as these can trigger the expression of the viral master lytic switch, replication, and transcription activator (RTA). RTA is a 691 amino acid protein that contains a transactivation domain, nuclear localization signal, DNA binding domain, and a RING-like domain and serves as the major transcription factor responsible for lytic gene expression. Once a threshold of RTA expression is met, viral lytic reactivation is triggered by induction of lytic gene expression [76,77]. During both latency and lytic reactivation, KSHV utilizes diverse methods of co-opting the host UPS to support the viral lifecycle (Table 2) [2,4,78].

KSHV begins modulating the host UPS prior to entry into the target cell. KSHV particles have been found to colocalize with and activate numerous isoforms of c-Cbl, a host E3 ligase that resides just beneath the plasma membrane [120,121]. Upon infection, KSHV induces rapid localization of c-Cbl to lipid rafts for macropinocytosis [79]. c-Cbl was responsible for monoubiquitinating integrins α3β1 and αVβ3, which direct macropinocytic entry, and polyubiquitinating and therefore degrading αVβ5, which would otherwise direct KSHV for clathrin-lysosomal non-infectious entry [79]. Additionally, c-Cbl has been discovered critical for clathrin-mediated endocytosis of KSHV as c-Cbl induces K63-polyubiquitination of EphA2, a crucial step for viral endocytosis to take place [80,81]. These findings are corroborated by the work of Dutta, et al., as they have demonstrated that c-Cbl knockdown reduced clathrin association with the virus in human foreskin fibroblasts (HFFs) [81].

#### 2.2.1. LANA

The virus establishes latency following primary infection, where LANA tethers the viral episome to the host chromatin [76]. LANA alone has been found to affect numerous cellular pathways by commandeering the host UPS. For example, LANA can disrupt host transcriptional activities early after infection by directly interacting with RLIM, a RING domain containing E3 ubiquitin ligase also known as RNF12, thereby enhancing its autoubiquitination and degradation [82]. RLIM is a negative regulator of LIM domain containing proteins, including transcription factors such as LMO2, TRF1, LHX3, and LDB1, which are either further ubiquitinated and degraded (LMO2 and LDB1) in response to LANA-RLIM interactions or protected from RLIM-induced degradation (LHX3 and TRF1) [82]. Furthermore, LANA indirectly influences chromosomal segregation and activity of the APC ubiquitin ligase complex via inhibition of BUB1 [83]. BUB1 is a serine/threonine kinase that functions in the spindle assembly checkpoint, ensuring correct chromosome positioning prior to chromosome segregation. The inhibition of BUB1 kinase activity leads to the predisposition of aneuploidy in part due to reduced Cdc20 phosphorylation leading to sustained APC activation, prompting premature exit from the cell cycle [83]. These indirect influences of LANA on the APC ubiquitin ligase complex provide a mechanism of how KSHV co-opting of the UPS can drive the cell cycle forward even while DNA segregation is encumbered, thus propelling oncogenesis and tumorigenic cell survival concomitantly [83].

LANA has also been demonstrated to downregulate numerous tumor suppressors. For example, Cai et al., describe how LANA recruits the EC5S ubiquitin complex to degrade the critical tumor suppressors p53 and von Hippel-Lindau (VHL), both of which are members of distinct E3 ubiquitin ligase complexes themselves [84]. Loss of these tumor suppressors enhances HIF-1α and Survivin activity thereby upregulating angiogenesis, cell proliferation, cell survival, and tumorigenesis [84,85]. LANA-induced degradation of p53 and VHL has been observed in both transfected and KSHV-infected B-lymphoma cell lines, suggesting a prominent role for LANA in stimulating oncogenesis [84,85,86]. Additionally, LANA has also been found to compete with intracellular Notch binding to the FBXW7 (Sel10) E3 ligase complex, thereby disrupting Notch polyubiquitination and subsequent degradation, further contributing to infected cell proliferation [86]. Together, these data suggest that LANA promotes cell proliferation and survival. LANA is therefore likely a major contributory source for the development of neoplastic effusions in the latency-associated malignancy, PEL.

#### 2.2.2. RTA

Upon environmental stressors, the latent-to-lytic transition can take place and KSHV RTA, the master lytic switch, will facilitate the lytic cascade of gene expression via its intrinsic transcriptional capabilities. However, viral modulation of the UPS is also necessary for the early onset of the latent-to-lytic transition. Our group has found that the human Itch ubiquitin ligase is necessary for RTA-mediated vFLIP degradation, a crucial event that leads to silencing of NFκB signaling and progression into lytic reactivation [104,105]. During this process, RTA, vFLIP, and the Itch-A20 ubiquitin editing complex come together, prompting RTA to induce the degradation of vFLIP [105]. NFκB is subsequently shut off, promoting lytic replication [104,105]. Interestingly, RTA also utilizes its intrinsic ubiquitin ligase capabilities to regulate its own abundance by auto-ubiquitinating itself for subsequent degradation [89].

KSHV RTA plays further dominant and diverse roles in modulating host cellular pathways during lytic reactivation, including disrupting critical mechanisms of innate immunity. One of the first lines of defense against invading pathogens is the toll-like receptors (TLRs), which function as part of the innate immune system to generate type 1 interferon production and NFκB-mediated inflammation in response to invading pathogens [90,91]. The Myeloid Differentiation Factor 88 (MyD88) is a critical cellular adapter utilized during TLR4 signaling for efficient induction of NFκB activation and has been found to be affected by RTA [90,91,92]. Particularly, Zhao et al. have demonstrated that RTA utilizes intrinsic E3 ligase capabilities to ubiquitinate MyD88, marking it for degradation by the proteasome [90]. As the primary function of RTA is to induce lytic reactivation, these findings are congruent with previous studies indicating NFκB signaling is enhanced by KSHV latent miRNAs and is required for mitigating lytic reactivation to maintain latency [91,105,122,123,124,125,126,127,128,129]. RTA has also been found to induce proteasome-mediated degradation of TRIF, another adaptor downstream of TLR signaling, further contributing to the downregulation of Type 1 Interferon [93,94,95]. However, TLR modulation via KSHV is not restricted to RTA. The HECT and RCC1-containing protein 5 (HERC5) is a ubiquitin ligase that functions downstream of TLR3 signaling for the ISGylation and/or ubiquitination of protein substrates [130,131]. ISG15 is a ubiquitin-like protein stimulated by type I interferons in response to pathogen invasion. ISG15 is conjugated to substrates and alters their stability or function via the process of ISGylation. The viral interferon regulatory factor 1 (vIRF1) is a lytic gene encoded by ORF K9 [132] and has been reported to interact with HERC5, resulting in decreased ISGylation of IRF3 [106]. It has been demonstrated previously that ISGylation stabilizes IRF3 by reducing its ubiquitination in a Pin1-dependent manner [133]. Therefore, vIRF1 attenuates IRF3 activity and reduces the type 1 interferon response normally mediated by TLR3 signaling [106,131,133].

In addition to crippling TLR signaling networks, RTA has other methods of shutting off Type I Interferon production and targeting lytic repressors [96,101,134]. A classic example of this is demonstrated in the work of Yu et al., where they demonstrated that RTA cooperates with the host E2 enzyme UbcH5A to directly polyubiquitinate IRF7 for proteasomal degradation [89]. Furthermore, this group describes RTA as increasing the stability of RTA-associated ubiquitin ligase (RAUL), which is known to facilitate proteasome-mediated degradation of IRF3 and IRF7 [102]. By utilizing its own E3 ligase activity and RAUL, RTA negatively regulates type I interferon production and innate immune defenses, thereby contributing to viral persistence and immune impairment [102,135]. Interestingly, RTA has also been found to directly interact with Hey1 and promote its ubiquitination and proteasomal degradation [96,97]. Hey1 is a mediator of Notch signaling as well as a positive regulator of p53 and a transcriptional repressor of the RTA promoter, suggesting that RTA facilitates a feedback loop of its own activation [96,136,137]. Further evidence indicates that KSHV induces downregulation of the inhibitor of DNA binding protein 2 (ID2) within four hours post-lytic reactivation [98,99]. It was later discovered that RTA reduces ID2 protein levels by direct binding and inducing its N-terminal ubiquitination and proteasomal degradation [98,99]. This modality of manipulation is important for KSHV, as ID2 and other ID family members are known to negatively regulate transcription factors associated with the progression of the cell cycle and the development of the immune system [99,138,139]. RTA intrinsic ubiquitin ligase activity is also required to degrade viral lytic repressors. For example, when the viral KSHV RTA-Binding Protein (KRBP) is at higher concentrations, it functions as an RTA transcriptional repressor [140,141] and RTA has been found to induce K-RBP degradation [101]. K8 (K-bZIP) is another viral protein demonstrated to bind RTA, limiting its transactivation functionality [142,143]. RTA counteracts this process by inducing K8 for degradation [101]. However, further studies are needed to confirm the degradation pathway and whether RTA is directly ubiquitinating K8 or whether RTA perhaps recruits a cellular ubiquitin ligase to facilitate its degradation [78,101].

KSHV employs additional mechanisms that contribute to lytic reactivation. The nuclear poly (ADP-ribose) polymerase 1 (PARP1) enzyme normally inhibits RTA from activating lytic gene promoters via direct interaction and poly (ADP-ribosyl)ation (PARylation) of RTA [107]. To counteract this, KSHV employs the early lytic gene product processivity factor 8 (PF-8), which recruits the host E3 ligase checkpoint with fork-head and ring finger domains (CHFR) [107]. A PF-8 homodimer forms and binds with CHFR, facilitating interaction between CHFR and its substrate PARP1 [107]. This interaction helps catalyze CHFR-mediated polyubiquitination and degradation of PARP1, relieving RTA PARylation, and thereby accelerating lytic replication [107]. PF-8 is also known to interact with the host UHRF1 E3 ubiquitin ligase, but further studies are needed to reveal any relation between this interaction with pathogenesis and viral persistence [107].

KSHV also employs the UPS in disrupting antigen presentation. RTA has been demonstrated to participate in modulating antigen presentation specifically via direct and indirect downregulation of HLA-DRα, an MHC-II α-chain paralogue, and membrane-bound RING-CH 8 (MARCH8) substrate [100,103]. MARCH E3 ligases are known to specifically target membrane-bound proteins for ubiquitination and internalization [144]. RTA has been shown to bind HLA-DRα directly, facilitating its direct ubiquitination by RTA, but also influences its indirect ubiquitination via RTA-mediated upregulation of MARCH8 expression, ultimately leading to HLA-DRα proteasomal degradation [100,103].

#### 2.2.3. K3 and K5

KSHV K3 and K5 are prototypic members of the membrane-bound RING-CH (MARCH) family of E3 ubiquitin ligases and K3 and K5 both downregulate MHC I molecules [109,110,144]. K5 is known to manipulate MHC-I upon entry into the lytic phase, whereas evidence suggests MHC-I modulation by K3 occurs later during the lytic replication [144]. Furthermore, K3-mediated ubiquitination has been established to be completely dependent upon an intact K3 RING domain [109,144,145]. K3 and K5 also demonstrate different MHC-I allotype specificities [109,110]. K3 downregulates allotypes HLA-A, -B, -C, and -E, whereas K5 downregulates only HLA-A and -B [109,110]. K3 and K5 specifically induce this downregulation via endocytosis and lysosomal-mediated degradation and not by interference with MHC-I synthesis [109,110,146,147]. These data are supported by work demonstrating that K3 induces specifically K63-linked polyubiquitination of MHC-I, leading to internalization and endolysosomal degradation [148,149]. K5 specifically downregulates the MHC-related chains (MIC)-A/B by inducing ubiquitination at their cytoplasmic tails, further leading to evasion from circulating CTLs and NK-cells [148,149,150]. Furthermore, K5-induced ubiquitination of MIC-A/B has been associated with downregulated cell surface MIC-A/B, but not internal degradation [150]. K3 and K5 modulation of immunoreceptors is not limited to MHC-I, as both K3 and K5 have been reported to downregulate cell surface γ-IFN receptor 1 (IFNG1) [111] and L-selectin [112]. L-selectin is a rolling/tethering receptor on monocyte and other leukocyte surfaces that aids in monocyte trans-endothelial migration [112,151]. The K3 and K5-mediated downregulation of L-selectin has been determined to take place via polyubiquitination of its cytoplasmic tail lysine residues and this polyubiquitination was also found to be dependent upon the viral RING domains in K3 and K5 [112]. However, K5 demonstrates higher promiscuity than K3, as it is known to downregulate additional immunoreceptors in addition to MHC-I, including CD86, ICAM1, Cd1d [113], CD31 (PECAM) [114], CD166 (ALCAM) [115], the activation-induced C-type lectin (AICL) [115], and vascular endothelial (VE)-cadherin [116]. Finally, K5 was reported to downmodulate Tetherin (BST2/CD317), an IFN-induced antiviral membrane protein that tethers budding virions to the cell surface, preventing viral propagation [117,118]. Tethered virions may then reside at the cell surface or be endocytosed and subsequently degraded. Tetherin is known to antagonize the budding of diverse viruses, including HIV-1, HIV-2, SIV, Ebola, and Influenza VLP’s [118,152]. Given the roles that immunoreceptors such as L-selectin and Tetherin play in the antiviral immune response, it is not surprising that they are frequent viral targets.

#### 2.2.4. Metabolic Reprograming

In addition to utilizing the host UPS to facilitate viral entry, enhance infected cell proliferation/survival, promote lytic onset, and evade immune processes, KSHV conducts a complete reprogramming of host cell metabolism. vIRF1 has been discovered to mediate cellular metabolism by recruiting the Kelch-like 3 (KLHL3) E3 ligase, which facilitates the formation of the KLHL3-Cullin-3 E3 ubiquitin ligase complex [108]. This complex mediates proteasomal degradation of the heterogeneous nuclear ribonuclear protein Q1 (hnRNP Q1), leading to reduced glycerophosphodiester phosphodiesterase domain containing 1 (GDPD1) mRNA expression, an associated increase in aerobic glycolysis, and the onset of the Warburg Effect [108]. This step is critical for KSHV survival as treatment with glycolytic inhibitors induced apoptosis in KSHV-infected endothelial cells [153]. K5 has also been associated with the induction of aerobic glycolysis, increased lactate production, and other contributory events to the Warburg Effect [119]. This, in part, takes place via altering endocytosis of the cellular growth factor-binding receptor tyrosine kinase and likely via upregulation of HIF-1α [119]. As a result, Akt activation and Erk1/2 phosphorylation are sustained, further driving cell proliferation and survival [119]. Although ubiquitination of a receptor by K5 was not observed, loss of the K5 ligase domain was not able to induce the same effects despite still interacting with targets. [119]. These data suggest a strong role of K5 ubiquitin ligase activity in the modulation of host cell metabolism, increased proliferation, and survival signaling [119].

KSHV has also evolved the ability to utilize the host UPS to disrupt the generation of reactive oxygen species (ROS). ROS are highly volatile molecules produced as natural byproducts of cellular metabolism that play critical roles in survival, apoptosis, cell differentiation, inflammatory responses, and other signaling processes [87,88]. The latent KSHV protein, kaposin A, has been found to trigger the upregulation of the HECT domain and ankyrin repeat containing E3 ubiquitin ligase protein 1 (HACE1), which blocks the Rac1-dependent NADPH oxidase-mediated generation of ROS. As excess ROS accumulation during viral infection can aid in apoptosis induction, this suggests Kaposin A-mediated HACE1 upregulation provides an additional means for KSHV to evade apoptosis and persist as a lifelong infection [87,88].

## 3. Human Immunodeficiency Virus

The Human Immunodeficiency Virus (HIV-1 and HIV-2) is a retrovirus that evolved from SIV. HIV is an enveloped virus with a genome comprised of two copies of positive sense single-stranded RNA and is the etiologic agent of acquired immunodeficiency syndrome (AIDS). While the development of effective antiviral therapies has had a significant impact on deaths resulting from HIV/AIDS, the share of deaths from this virus and associated infections remains as high as 1 in 4 in Southern Sub-Saharan Africa. Globally, it is estimated that more than 36 million people are living with HIV [154].

Following exposure to the virus, the virion, containing the RNA genome bound by host tRNA and nucleocapsid protein encased in a lattice of capsid protein, covered with matrix, and coated in a host-derived membrane containing envelope protein (gp120) trimers, binds to host cells containing CD4 and CCR5 or CXCR4. Consequently, the cell tropism for this virus is primarily CD4-positive helper T cells and macrophages, and untreated infection results in immunodeficiency as the adaptive arm of the immune system is incapacitated [154].

The HIV genome is capped at the 5′ end and polyadenylated at the 3′ end and organized into the gag, pol, and env genes flanked by repeats, untranslated regions, and a 5′ primer binding site and packaging signal. In addition to the typical retrovirus genome organization described above, HIV also encodes auxiliary genes that function to interact with and counteract host processes and defenses [154]. One way in which this virus, like all viruses, has evolved to co-opt and control the host cellular machinery is via hijacking the UPS (Table 3). There have been many reviews on ubiquitin and ubiquitin-like proteins in HIV infection; here, we will limit our discussion to viral interaction with E3 ubiquitin ligases (often Cullin ubiquitin ligases) [155,156,157,158,159,160,161,162,163].

The binding of gp120 to CD4 and CCR5/CXCR4 results in virion/host membrane fusion and the matrix and capsid-coated genomes are released into the cytoplasm. The viral genome is reverse transcribed, and the pre-integration complex travels on microtubules to the nucleus where it is imported through nuclear pores, and the provirus is integrated into the host genome. Host transcription factors initiate early gene expression. Nef, Tat, and Rev are transcribed, spliced, exported from the nucleus, and translated. Tat is a transcription enhancer that binds to the transactivation response element (TAR) within nascent transcripts and promotes phosphorylation and processivity of RNA polymerase and Rev binds intron containing genomes and transcripts and promotes their export from the nucleus [154].

### 3.1. Nef

Nef, which stands for Negative regulatory factor, was originally identified as a negative regulator of HIV replication in some HIV-1 and HIV-2 subtypes [194]. It was later found that this myristolated protein interacts with membranes and plays a role in the downregulation of multiple cell surface receptors, including CD4, CD28, CXCR4, and MHC class I and II, by inducing endocytosis [195,196,197,198,199,200]. This reduces superinfection, enhances virion release, and promotes immune evasion. Nef was shown to recruit the HECT domain E3 ligases AIP4 or NEDD4 to induce the endocytosis and degradation of CXCR4 and additional chemokine receptors via the ESCRT pathway via both ubiquitin-dependent and independent mechanisms [164].

Nef also counteracts the SERINC3/5 restriction factors, increasing the infectivity of nascent virions [165,166]. SERINC 3 and 5 are members of the serine incorporator protein family and are thought to function in the incorporation of serine into phosphatidylserine and sphingolipids; however, they are best characterized as lentivirus restriction factors. SERINC3/5, in the absence of Nef, is incorporated into newly assembled virions and restricts virus replication at the entry stage of virus-cell membrane fusion, however the exact mechanism of restriction is not fully understood [165,166]. Nef antagonizes this restriction factor, targeting SERINC5 to endosomes/lysosomes for degradation. Nef targets a cyclinK/Cdk13 kinase complex to phosphorylate SERINC5 to promote interaction with the AP2 adaptor complex, which targets the restriction factor for Nef-dependent endocytosis and degradation via a ubiquitin-dependent mechanism [167].

Nef is also reported to target p53 for ubiquitination and degradation via the recruitment of the cellular ubiquitin ligase E6AP; this observation supports earlier claims that Nef binds p53 and inhibits p53-mediated apoptosis [168,169].

### 3.2. HIV Hijacks Cullin Ubiquitin Ligases

Rev, by aiding the export of unspliced and singly spliced transcripts from the nucleus, promotes late gene expression. Gag and gag-pol are translated from the unspliced transcripts and Vif, Vpr, Vpu, and Env are translated from the singly spliced transcripts. Vif, Vpr, Vpu, and Vpx are all reported to hijack the ubiquitin–proteasome system, often interacting with Cullin RING Ubiquitin ligases (CRL) to evade host defenses [161]. CRLs are modular ubiquitin ligases that utilize a Cullin (1, 2, 3, 4, or 5) scaffold upon which a substrate adaptor and substrate receptor assemble. The substrate receptor binds to the substrate, bringing it in close proximity to the E2, which is bound to the Cullin scaffold via Rbx1/2. CRLs are widely hijacked by diverse viruses. Paramyxovirus, Adenovirus, Hepatitis B virus, herpesvirus, rotavirus, HIV-1, Hepatitis E virus, and more have been reported to co-opt CRLs [161].

#### 3.2.1. Cullin 1 CRL

Vpu assembles with the Cul1 substrate receptor β-TrCP to target multiple host proteins, most notably tetherin, and CD4. Vpu is an accessory protein that is unique to HIV-1. The gene encodes a transmembrane protein that is phosphorylated on its cytoplasmic domain by casein kinase-2 [201]. This phosphorylation mediates interaction with the Cul1 substrate receptor β-TrCP [171]. It is via this interaction that Vpu targets tetherin (BST-2), CD4, SNAT1, and PSGL-1 [170,171,172,173]. Tetherin is a type-I interferon-induced restriction factor that is expressed in B and T, dendritic, and myeloid cells. This membrane protein is localized to lipid rafts within the endomembrane system, cycling between plasma membrane, cell surface, and trans-Golgi network. In the presence of tetherin, Vpu negative virus can bud normally from the plasma membrane but remains tethered to the cell surface [170]. Tetherin functions as a physical tether by binding to nascent virions and preventing release from the infected cell, literally tethering viral particles to the host membrane and restricting the release of the cell-free virus. Tetherin also links virions to each other. This mechanism of host restriction has been demonstrated in multiple viruses including KSHV (described in this review). Tetherin is also reported to activate a pro-inflammatory response via NF-κB signaling. Tetherin becomes incorporated into the viral envelope as it buds from the host membrane and interacts with cell membrane-bound tetherin, preventing release from the virus-producing cell [202,203]. Vpu counteracts this activity via the recruitment of tetherin to Cul1-β-TrCP ubiquitin ligases and subsequent ubiquitin-dependent sequestration and/or downregulation of the restriction factor via proteasomal and/or lysosomal degradation [170]. SIV Nef degrades tetherin by recruiting the same Cul1-β-TrCP complex [204].

Vpu also targets cell surface protein and viral receptor CD4 for ubiquitination and degradation via the Cul1-β-TrCP ubiquitin ligase [171,205]. Downregulation of CD4 occurs via endoplasmic reticulum-associated degradation (ERAD) and promotes nascent virion release by preventing binding of viral envelope to CD4 during virion egress [171]. Proteomics-based studies have identified additional targets of the Vpu-Cul1-β-TrCP ubiquitin ligase including (but not limited to) SNAT-1, PSGL-1, ICAM-1, and 3, HLA-C, NTB-A, CD1d, PVR, CCR7, and CD62L [172,173,206,207,208,209,210,211,212,213]. Many of these targets have immunomodulatory and metabolic effects. SNAT-1 is an amino acid transporter that is degraded via lysosome and is thought to play a role in establishing virus reservoirs; however, the mechanism is not completely understood [173]. PSGL-1 is a cell surface glycoprotein that binds to selectins and functions in tethering and migration of CD4+ T cells into tissues. PSGL-1 blocks the binding of viral particles to target cells [174]. Vpu and Nef expression counteracts inhibition by PSGL-1 by downmodulating the protein from the cell surface [172]. PSGL-1 is thought to have broad antiviral activity as it was shown to inhibit diverse viruses murine leukemia virus and influenza A virus [174].

#### 3.2.2. Cullin 4 CRL (Cullin Ring Ubiquitin Ligase)

Vpx and SIV Vpr assemble with DCAF1 (also known as VPRBP) a substrate receptor for Cullin 4 CRL (Cullin Ring ubiquitin ligase) and target SAMHD1 for degradation. SAMHD1 is a restriction factor that inhibits HIV-1 infection of nondividing cells of myeloid lineage and resting CD4+ T cells [214,215,216]. SAMHD1 is a deoxynucleoside triphosphate (dNTP) hydrolase that cleaves the triphosphate from the deoxynucleotide [217,218,219]. In the presence of dGTP, SAMHD1 hydrolyzes all dNTPs, acting to deplete the pool of cellular nucleotides required for reverse transcription (RT) of viral RNA to DNA [220]. Reverse transcription is an essential step in the lentiviral lifecycle and SAMHD1 effectively inhibits RT and viral replication [220]. Vpx, encoded by HIV-2 and some lineages of SIV, counteracts the activity of SAMHD1 by targeting the restriction factor for proteasomal degradation [175,176,215,221]. Vpx and SIV Vpr assemble with DCAF1 (also known as VPRBP) a substrate receptor for Cullin 4 CRL (Cullin Ring ubiquitin ligase) and target SAMHD1 for degradation, allowing for productive infection to occur [175,176,177]. Assembly of Vpx with the DCAF1 CRL4 is dependent on a zinc coordinated structure, as zinc chelation with N,N,N’,N’-Tetrakis(2-pyridylmethyl)ethylenediamine (TPEN) inhibits SAMHD1 degradation [222]. The interaction of Vpr and Vpx with DCAF1 is dependent upon a conserved motif that is required for Vpx degradation of SAMHD1 and Vpr-induced cell cycle arrest [223]. It is hypothesized that Vpx arose from the duplication of an ancestral Vpr, and each gene evolved divergent functions [224,225]. Ancestral Vpr evolved the SAMHD1 antagonizing features that were maintained in Vpx but were lost from HIV-1 Vpr over millions of years of evolution. This could explain why HIV-2 and some SIV retain Vpx or Vpr that can counteract SAMHD1, while HIV-1 does not and is susceptible to SAMHD1 activity, remaining a restriction factor for infection in certain cell types.

HIV-1 Vpr targets MCM10, UNG2, SMUG, Dicer, and telomerase via the Cul4-DDB1 E3 ubiquitin ligase. HIV-1 Vpr is known to interact with the CUL4A-RBX1-DDB1-DCAF1/VPRBP E3 ubiquitin ligase and this interaction is required for cell cycle arrest in G2 [226,227,228,229,230,231,232]. In fact, DCAF1 is also known as VprBP, due to this well-characterized interaction. Interaction with this ubiquitin ligase is known to promote the degradation of MCM10, UNG2, SMUG, Dicer, TET2, and telomerase [178,180,181,182,183,184,233,234]. Unlike other viral proteins that function to recruit substrates to ubiquitin ligases, Vpr acts to enhance the degradation of known substrates of the Cul4-DDB1 ubiquitin ligase [181,182,183,184]. While Vpr is known to promote G2/M arrest via interaction with SLX4 and subsequent activation of the DNA damage response, Vpr enhanced degradation of MCM10 was also reported to be associated with promoting G2/M cell cycle arrest [178,179]. UNG2 and SMUG are uracil DNA glycosylases that repair uracil-containing DNA following C→U deamination, an outcome of APOBEC3F and G activity. Vpr targets these enzymes for ubiquitination and proteasomal degradation, increasing ΔVif virus infectivity even in viruses containing low levels of A3F and A3G [180]. Vpr was shown to target endoribonuclease Dicer via Cul4 as well to counteract the inhibitory effects of this enzyme on HIV-1 infection of macrophages [182]. Telomerase is responsible for adding telomere sequences to the ends of chromosomes. Vpr was found to downregulate telomerase activity by targeting TERT, the catalytic subunit of the telomere-lengthening enzyme, for proteasomal degradation [183,184]. Vpr enhances the interaction between TERT and DCAF1, therefore promoting TERT ubiquitination and degradation [183,184]. This accounts for the previously observed decreased telomerase activity in HIV-1-infected peripheral blood mononuclear cells [235,236].

#### 3.2.3. Cullin 5 CRL

Vif induces the ubiquitination and degradation of APOBEC3G/F (A3G/F) and STAT1/3 via the Cullin 5 CRL (CRL5). The APOBEC3 proteins are a family of DNA editing enzymes that are encoded in tandem on chromosome 22 [237]. A3G was first identified in a screen for restriction factors. Two nearly identical cell lines (CEM and CEM-SS) were infected with Vif deficient HIV-1 virus and only one of the cell lines (CEM-SS) was permissive for infection [238,239]. It was later found that this difference was due to the presence of A3G [240]. In the absence of HIV Vif, A3G, a cytidine deaminase, is packaged into virions and upon infection of a new cell, deaminates cytosine to uracil, resulting in viral genome hypermutation [241,242,243]. This significantly reduces the production of infectious virions. The virus evolved to counteract this host restriction factor via the activity of Vif. Vif excludes A3G from virions by acting as a substrate receptor, along with CBFβ, for a Cul5 CRL [185,186,187,188,189,190,191,192]. Vif interacts with Cul5, A3G (and A3F), CBFβ, and substrate adaptor Elongin C to counteract this restriction factor, effectively excluding it from virions [192]. Interaction with CBFβ also has the effect of inhibiting the natural binding partner, the RUNX transcription factor, decreasing immune response-related gene expression [192].

Vif has also been reported to target the IFN-induced Jak–STAT pathway via the Cul5 CRL. Vif assembles with Cul5 to target STAT1 and STAT3 for proteasomal degradation and this is dependent upon the SOCS box motif in Vif, Cul5, and Rbx2 [193]. This pathway is a frequent target of viruses as it plays a significant role in the antiviral response.

## 4. Conclusions

Viruses have evolved diverse mechanisms to ensure successful infection, persistence, and replication. Here, we have focused on how EBV, KSHV, and HIV ensure lifecycle success via hijacking of the host UPS. These viruses employ diverse programs and processes to commandeer this intricate system, including the upregulation of the recruitment of host E3 ligases, inducing the editing of host E3 ligase substrate ubiquitination, and utilizing virally encoded E3 ligases to disrupt critical cellular networks. In KSHV, one of the critical players in this modulation is the master lytic switch, RTA, whose homologs in EBV and murine gammaherpesvirus 68 (MHV-68) demonstrate functionally conserved evolutionary roles [99]. For example, KSHV, EBV, and MHV-68 RTA homologs interact with ID2 and induce the degradation of all four members of the ID protein family [99]. Modulation of these proteins has been linked to different cancers, including breast cancer, prostate cancer, and colorectal cancer [99,244]. Additionally, K3, K5, and mK3, a K3 homolog from MHV-68, have all been found to downregulate the expression of MHC I molecules [109,110,144]. It will therefore be no surprise to discover additional evolutionarily conserved modalities between oncogenic herpesviruses and the human UPS. Likewise, HIV has evolved a preference for CRLs, encoding accessory proteins that interact with substrate receptors, recruiting host restriction factors and antiviral proteins thereby targeting them for polyubiquitination and proteasomal degradation.

The interactions between viruses and the UPS represent a source of therapeutic opportunity. The fact that a virus has evolved to target specific proteins and processes, suggests that this is a point of weakness that should be targeted for intervention. Currently, there are many FDA-approved proteasome and immunoproteasome inhibitors that are being used in the clinic as well as a number of inhibitors of E1s, E2s, and E3s [245]. As reviewed by Pei et al., many inhibitors of the proteasome have demonstrated effectiveness in the treatment of EBV-associated cancers, which provides support for UPS inhibition as a strategy for antiviral therapy [17]. Indeed, the first FDA-approved proteasome inhibitor, bortezomib, has been evaluated in numerous viral contexts. Importantly, a more recent study found that its use in both KSHV- and EBV-infected cells triggered lytic reactivation providing clear evidence for how targeting the UPS system could limit viral spread [246]. More of these inhibitors and inhibitors of E1, E2, and E3s can be evaluated for effectiveness against viruses where targeting of host processes is critical for viral infection, replication, and/or disease establishment/progression.

Few FDA-approved E3 targeted inhibitors would be useful for the viruses covered in this review and this represents an area with potential for advancement. A possible example is Skp2 inhibitors, which are an active area of research in cancer therapeutics and should be evaluated for anti-HIV activity [245]. Very recently, the HECT E3 ligase inhibitor, I3C, was evaluated for SARS-CoV-2 and was able to potently inhibit the virus and reduce viral egress [247]. I3C is a natural inhibitor of NEDD4 and WWP1 [247] so it is possible that this may also be effective in HIV, which also utilizes NEDD4. Additionally, Deschamps et al. were recently able to identify a novel inhibitor targeting ICP0, an HSV1 E3 ligase, by developing a high-throughput screening technique based on autoubiquitination of ICP0 [248]. It is therefore possible that a similar technique may be used to identify inhibitors of other viral E3 ligases including, for example, RTA, K3, or K5 in KSHV. On a final note, PROteolysis Targeting Chimeras or PROTACs is an emerging field of study for antiviral therapy and may represent a novel way for targeting UPS-interacting viral proteins. PROTAC technology utilizes cellular UPS machinery to degrade desired target proteins and has begun to be examined for antiviral and vaccine potential in multiple viruses including HIV (reviewed by Ahmad et al.) [249]. This represents a potential unique twist on other UPS inhibitors as they are designed to utilize intact host UPS networks. It is possible that PROTAC technology could be used to design a targeted therapy for viral E3 ligases or viral proteins that critically target the UPS, and this may be aided by the already close interactions we reviewed above. This highlights the importance of understanding the role of the UPS in the viral life cycle and the need for further study into the identification and evaluation of UPS inhibitors as antiviral therapy.

## Figures and Tables

**Table 1:** E3 ubiquitin ligase interactions in EBV.

Phase	Viral Protein	Ligase	Target Protein	Effect	Reference(s)
Latent	LMP1	TRAF6	IKK	Activate NFκB to maintain viral latency	[18,19,20,21,22]
			p38	Activate MAPK signaling to disrupt BCR signaling	[18]
		LUBAC	NEMO	Activate NFκB to maintain viral latency	[23]
			IRF7	Activate NFκB to maintain viral latency	[23]
		MDM2	p53	Promote p53 activation to aid cell survival	[24]
		TRAF2	p53	Promote p53 activation to aid cell survival	[24]
		CHIP	RIG1 *	Reduce IFNβ expression * to aid immune evasion	[25]
	LMP2A	AIP4	Lyn	Reduce BCR signaling to aid immune evasion	[26,27,28,29,30]
			Syk	Reduce BCR signaling to aid immune evasion	[26,27,28,29,30,31]
		WWP2	Lyn	Reduce BCR signaling to aid immune evasion	[26,27,28,29,30]
			Syk	Reduce BCR signaling to aid immune evasion	[26,27,28,29,30,31]
		Cbl-b	Syk	Reduce BCR signaling to aid immune evasion	[31,32]
		Itch	LMP2A	Reduce EBV signaling to aid in host defense	[27]
		c-Cbl	LMP2A	Reduce EBV signaling to aid in host defense	[33]
	EBNA1	STUB1	KAP1 ^+^	Regulate latent to lytic switch	[34]
	EBNA3C	SCF^skp2^	Rb	Dysregulate the cell cycle	[35,36]
			p27	Dysregulate the cell cycle	[36,37]
		MDM2	p53	Dysregulate the cell cycle	[38]
Lytic	BPLF1	TRIM25	TRIM25	Reduce type 1 interferon response to aid immune evasion	[39]
	BGLF2	Cul1	STAT2	Reduce innate immune response to aid immune evasion	[40]
	BFRF1	Itch	BFRF1	ESCRT-associated vesicle formation for virion release	[41,42]
	BRLF1	TRIM5α	BRLF1	Mediate lytic reactivation	[43]

* Proposed but unconfirmed; ^+^ SUMOylation.

**Table 2:** E3 ubiquitin ligase interactions in KSHV.

Phase	Viral Protein	Ligase	Target Protein	Effect	Reference(s)
Entry	Viral Particle	c-Cbl	Integrins	Activate micropinocytosis for viral entry	[79]
			EphA2	Activate clathrin-mediated endocytosis for viral entry	[80,81]
Latent	LANA	RLIM	RLIM	Modulate host transcriptional machinery	[82]
			LMO2	Reduce host transcription	[82]
			LDB1	Reduce host transcription	[82]
			TRF1	Activate host transcription	[82]
			LHX3	Activate host transcription	[82]
		APC Complex		Promote exit from the cell cycle	[83]
		EC5S	p53	Promote angiogenesis, cell proliferation, and cell survival	[84,85]
			VHL	Promote angiogenesis, cell proliferation, and cell survival	[84,85]
		FBXW7	Notch	Promote cell proliferation	[86]
	Kaposin A	HACE1	Unknown	Block production of ROS and subsequent apoptosis	[87,88]
Lytic	RTA	RTA	RTA	Mitigate lytic reactivation	[89]
			MyD88	Reduce NFκB activity to promote lytic reactivation	[90,91,92]
			TRIF	Reduce type 1 interferon response to aid immune evasion	[93,94,95]
			IRF7	Reduce type 1 interferon response to aid immune evasion	[89]
			Hey1	Increased RTA transcription	[96,97]
			ID2	Dysregulate the cell cycle and host immune response to aid immune evasion	[98,99]
			HLA-DRα	Reduce antigen presentation to aid immune evasion	[100]
			KRBP	Propagate RTA signaling	[101]
		RAUL	IRF7	Reduce type 1 interferon response to aid immune evasion	[102]
			IRF3	Reduce type 1 interferon response to aid immune evasion	[102]
		MARCH8	HLA-DRα	Reduce antigen presentation to aid immune evasion	[100,103]
		Itch	vFLIP	Reduce NFκB signaling to promote lytic reactivation	[104,105]
		HERC5	vIRF1 ^x^	Reduce type 1 interferon response to immune evasion	[106]
		Unknown	K8	Reduce transactivation	[101]
	PF-8	CHFR	PARP1	Reduce PARylation of RTA to accelerate lytic replication	[107]
		UHRF1	Unknown	Unknown	[107]
	vIRF1	KLHL3	hnRNP Q1	Activate aerobic glycolysis and lactate production to induce the Warburg Effect	[108]
	K3	K3	HLA-A	Reduce antigen presentation to aid immune evasion	[109,110]
			HLA-B	Reduce antigen presentation to aid immune evasion	[109,110]
			HLA-C	Reduce antigen presentation to aid immune evasion	[109,110]
			HLA-E	Reduce antigen presentation to aid immune evasion	[109,110]
			IFNG1	Reduce interferon response to aid immune evasion	[111]
			L-selectin	Reduce monocyte endothelial migration *	[112]
	K5	K5	HLA-A	Reduce antigen presentation to aid immune evasion	[109,110]
			HLA-B	Reduce antigen presentation to aid immune evasion	[109,110]
			IFNG1	Reduce interferon response to aid immune evasion	[111]
			L-selection	Reduce monocyte endothelial migration *	[112]
			CD86	Reduce host immune response to aid immune evasion	[113]
			ICAM1	Reduce host immune response to aid immune evasion	[113]
			Cd1d	Reduce host immune response to aid immune evasion	[113]
			CD31	Reduce host immune response to aid immune evasion	[114]
			CD166	Reduce host immune response to aid immune evasion	[115]
			AICL	Reduce host immune response to aid immune evasion	[115]
			VE-cadherin	Reduce host immune response to aid immune evasion	[116]
			Tethrin *	Reduce host immune response to aid immune evasion	[117,118]
			Unknown	Modulate host metabolism to induce the Warburg Effect	[119]

* Proposed but unconfirmed, ^x^ ISGylation.

**Table 3:** E3 ubiquitin ligase interactions in HIV.

Viral Protein	Ligase	Target Protein	Effect	Reference(s)
Nef	AIP4	CXCR4	Disrupt T cell development to aid immune evasion	[164]
	NEDD4	CXCR4	Disrupt T cell development to aid immune evasion	[164]
	Unknown	SERINC5	Promote viral infectivity	[165,166,167]
	E6AP	p53	Reduce apoptosis	[168,169]
Vpu	β-TrCP	Tethrin	Reduce viral restriction	[170]
		CD4	Promote virion release	[171,172]
		SNAT1	Establish virus reservoirs *	[173]
		PSGL-1	Promote viral infectivity	[172,174]
Vpx	DCAF1 (Cul4 CRL)	SAMHD1	Promote productive viral infection	[175,176,177]
Vpr	Cul4-DDB1	MCM10	Promote G2/M cell cycle arrest	[178,179]
		UNG2	Promote viral infectivity	[180,181]
		SMUG	Promote viral infectivity	[180]
		Dicer	Reduce viral restriction	[182]
		TERT	Reduce telomerase activity	[183,184]
Vif	CRL5	A3G/F	Reduce viral restriction	[185,186,187,188,189,190,191,192]
		STAT1	Reduce antiviral response	[193]
		STAT3	Reduce antiviral response	[193]

* Proposed but unconfirmed.
